# Improved Ablative Properties of Nanodiamond-Reinforced Carbon Fiber–Epoxy Matrix Composites

**DOI:** 10.3390/polym13132035

**Published:** 2021-06-22

**Authors:** Umar Farooq, Muhammad Umair Ali, Shaik Javeed Hussain, Muhammad Shakeel Ahmad, Amad Zafar, Usman Ghafoor, Tayyab Subhani

**Affiliations:** 1 Department of Material Science and Engineering, Institute of Space Technology, Islamabad 44000, Pakistan; umr.frq85@gmail.com; 2 Satellite Research & Development Centre, SUPARCO, Lahore 54590, Pakistan; 3 Department of Unmanned Vehicle Engineering, Sejong University, Seoul 05006, Korea; umair@sejong.ac.kr; 4 Department of Electrical and Electronics, Global College of Engineering and Technology, Muscat 112, Oman; 5 UM Power Energy Dedicated Advanced Centre (UMPEDAC), Higher Institution Centre of Excellence (HICoE), Level 4, Wisma R&D, University of Malaya, JalanPantai Baharu, Kuala Lumpur 59990, Malaysia; shakeelalpha@gmail.com; 6 Department of Electrical Engineering, University of Lahore, Islamabad 44000, Pakistan; amad.zafar@ee.uol.edu.pk; 7 Department of Mechanical Engineering, Institute of Space Technology, Islamabad 44000, Pakistan; 8 Department of Mechanical Engineering, College of Engineering, University of Ha’il, Ha’il 81451, Saudi Arabia

**Keywords:** nanodiamonds, thermal conductivity, ablation, erosion rate

## Abstract

The influence of nanodiamonds (NDs) on the thermal and ablative performance of carbon-fiber-reinforced–epoxy matrix compositeswas explored. The ablative response of the composites with 0.2 wt% and 0.4 wt% NDs was studied through pre-and post-burning morphologies of the composite surfaces by evaluation of temperature profiles, weight loss, and erosion rate. Composites containing 0.2 wt% NDs displayed a 10.5% rise in erosion resistance, whereas composites containing 0.4 wt% NDs exhibited a 12.6% enhancement in erosion resistance compared to neat carbon fiber–epoxy composites. A similar trend was witnessed in the thermal conductivity of composites. Incorporation of composites with 0.2 wt% and 0.4 wt% NDs brought about an increase of 37 wt% and 52 wt%, respectively. The current study is valuable for the employment of NDs in carbon fiber composite applications where improved erosion resistance is necessary.

## 1. Introduction

High-speed moving objects at high altitude dissipate energy in the form of heat. A part of the heat interacts with the surface of the object through convection or the radiation process. The thermal protection system (TPS) on the surface is designed to manage this high-heat energy and hence deliver protection to the structure and sensitive electronics equipment inside [1].

**Figure 1:**
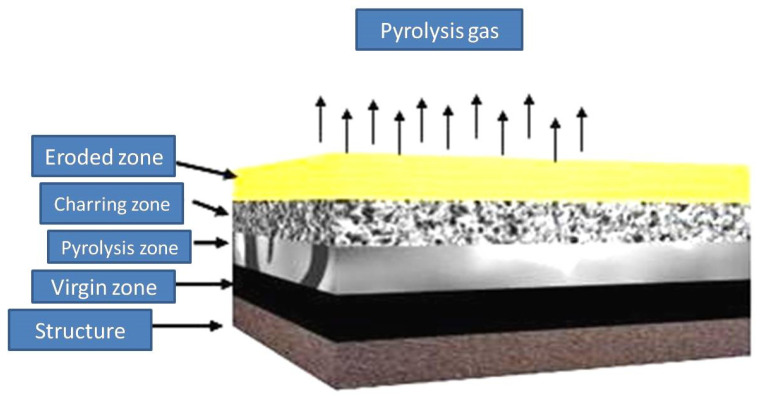
Schematic diagram of the mechanism of ablation of thermosetting resins.

Carbon-based materials as ablative materials can be considered for TPS use due to their remarkable mechanical and thermal properties [2,3,4]. Carbon fiber (CF) reinforced epoxy composite can be a good candidate as an ablator that sustains its reliability till sublimation [5]. TPSs are also being designed and tested for thermosetting polymers that are flame retardants [6]. In principle, high temperatures decompose (pyrolyze) the material, and as the diffusion of heat rises, the pyrolysis zone proceeds into the bulk material and the disintegration starts on the outer surface and discharges gaseous products, leaving carbonaceous residue. The gaseous products diffuse the porous char to the surface and absorb energy from char while enduring further decomposition, including cracking. These products finally exit into the boundary layer, where they may endure an additional chemical reaction with the boundary-layer gases [7]. A schematic description of the mechanism of ablation is presented in Figure 1. Four zones, i.e., (1) the virgin zone, (2) pyrolysis zone, (3) charring zone, and (4) eroded zone, can be seen from top to bottom of the sacrificial coating over the surface that needs to be protected. The charred products are fragile and can be removed by the mechanical thrust of gases coming out from the rocket engine combustion chamber or atmospheric erosion for re-entry vehicles, thus reducing the efficiency of these ablators. Different fillers like metal oxides, carbides, and/or glasses are being utilized to address this problem [8,9,10]. There are sometimes severe thermal conditions that cause the high mechanical erosion of matrix material. Limitations, like the production of mechanically weak char, rigorously reduce the lifespan of insulators, demanding additional dead weight.

Nanofillers in composites have also been considered, owing to their enhanced thermal resistance properties. Layered silicates as well as carbon micro-and nanofillers (carbon nanotubes) [11,12] are considered next-generation ablative materials. Other nano-fillers appropriate for ablative composites can be surface-treated montmorillonitenanoclays, carbon nanofibers, nanosilicon carbide whiskers, halloysite nanotubes, nano-graphene platelets, perovskite compounds, nanosilica, nanoalumina, polyhedral oligomericsilsesquioxane, and nanosilicon carbide particles [10,13]. Recently, a study was conducted to explore the influence of multi-walled carbon nanotubes (MWCNTs) on the ablative performance of CFRE-Cs [14]. Scanning electron microscopy (SEM) images and the thermal conductivity analysis of pre- and post-burnt samples with thermogravimetric analysis (TGA) and oxyacetylene flame displayed that MWCNTs in the matrix amplified its thermal conductivity, which enhanced the erosion resistance.

The current study was executed on CFRE-Cs utilizing different percentages of NDs as filler under extreme conditions. An allotropic form of carbon has been used for various optoelectronic, thermal, and decorative applications [15,16]. Thermogravimetric analysis was employed to evaluate the thermal stability of the composites. The erosion rate was calculated using a hyperthermal environment simulated by using an oxyacetylene flame arrangement. Insulation-to-density properties and insulation indexes were evaluated by utilizing oxyacetylene torch test result data. Thermal conductivity was measured to estimate the influence of NDs on CFRE-Cs.

Scanning electron microscopy was performed to analyze the pre- and post-thermal conditions of composites with varying NDs content.

## 2. Experimental Setup

**Figure 2:**
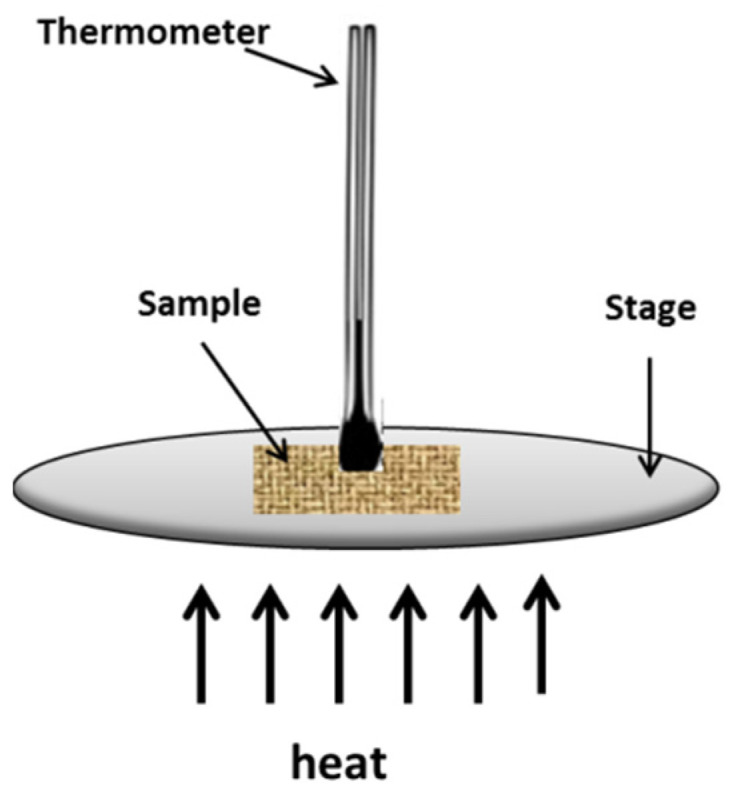
Thermal conductivity test arrangement.

The CFRE-Cs, with and without reinforcement of NDs, were manufactured by employing the vacuum-assisted resin transfer molding (VARTM) technique. The resultant laminates comprised ~50 wt% 3k-plain weave CFs and ~50 wt% aerospace-grade Araldite^®^ 5052 epoxy resin as a matrix material. The matrix material comprising functionalized NDs [17] dispersed in the epoxy matrix was infused into the CF architecture using a vacuum bagging assembly connected to a vacuum pump. After being cured at room temperature for 24 h, these laminates were de-molded and post-cured in an oven at 100 °C for 4 h. Afterward, the density of the manufactured CFRE-Cs was measured by using a densimeter (A&D Japan GF-300) working on the Archimedes principle. To measure the thermal conductivity performance of CFRE-Cs, with and without NDs, specimens were cut in a rectangular shape with dimensions of (7 mm × 7 mm × 1 mm) as suggested in the ASTM D5930 standard [18]. A hot plate was utilized to serve as an electric heating platform, and to measure the rise in temperature over time a thermometer was used, as displayed in Figure 2. Five specimens of each CFRE-C were used to record the thermal response at 80 °C and later the thermal conductivity values were calculated according to the standard procedure.

The as-received detonation-synthesized NDs were first treated with ozone and functionalized with phenylphosphonicacid (PPA) before mixing with the epoxy matrix. In brief, a 2 g ND powder was placed in a flask and 20 mL of dichloromethane was added dropwise to avoid any effervescence. Afterward, ozone was introduced at a 1 L/m flow rate and the process continued for 5 h. The oxidated NDs were then treated with PPA for surface functionalization. The PPA to NDs concentration was 3:5. Di-ionized water was used for dilution and the mixture was stirred for 48 h at 80 °C. Afterward the suspension was centrifuged at 6000 rpm and dried overnight at 60 °C.

The thermal analysis of the composite samples was conducted using a Mettler-Toledo, TGA/DSC 1 instrument. The critical analysis was performed using a temperature range from 25 °C to 1200 °C at a heating rate of 15 °C/min. For reference, thermogravimetric analysis of the components utilized to manufacture the CFRE-Cs, i.e., neat epoxy matrix, CF, and NDs, were also performed.

**Figure 3:**
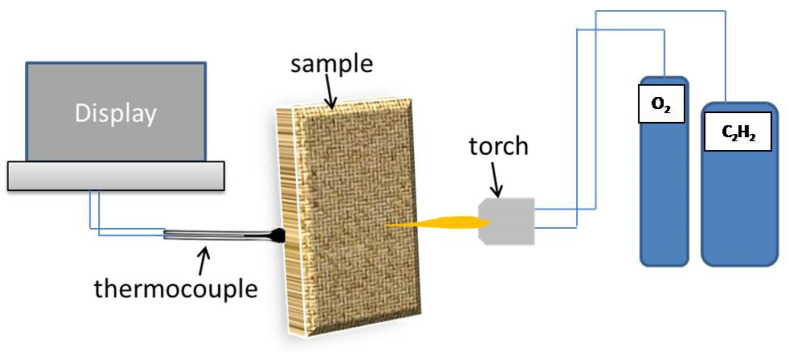
Oxyacetylene torch apparatus used to perform ablation tests.

**Figure 4:**
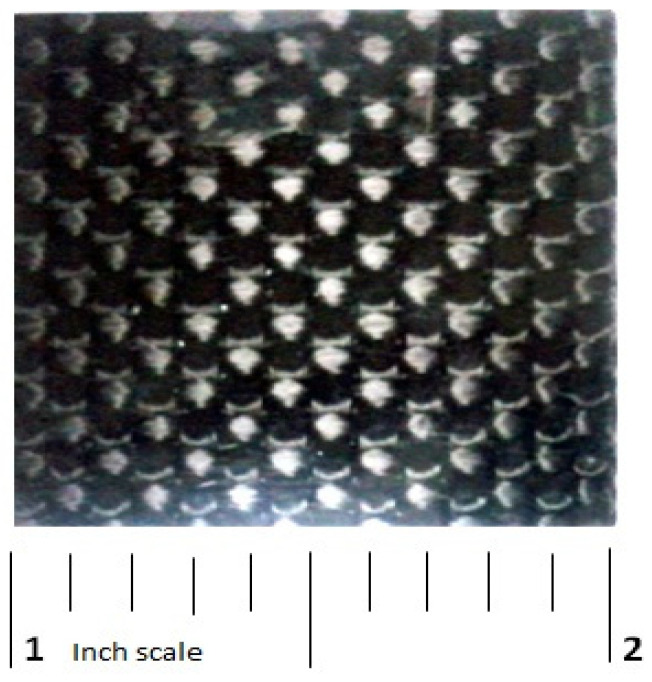
Composite specimen used for ablation tests.

The oxyacetylene torch apparatus, presented in Figure 3, was employed to perform ablation tests of the manufactured CFRE-C specimens with and without incorporation of NDs according to the ASTM E285-80 standard [19]. CFRE-C specimens having dimensions of 25 mm × 25 mm × 1 mm were obtained, as presented in Figure 4, and firmly held in place before exposure to the oxyacetylene flame for acquiring ablation test results. To measure the temperature of the opposite side of the CFRE-C specimens, a K-type thermocouple was used. A neutral oxyacetylene flame was then brought nearer to the specimens to perform an ablation test until burn-through of the CFRE-C specimens was reached. The utilized neutral flame was attained by physically adjusting the flow controls to 200 m/s velocity, 830 W/cm^2^ heat flux value, and 3000 °C temperature [19]. Field-emission scanning electron microscopy (FESEM) MIRA3 TESCON was utilized to observe and explore the microstructural alterations of composite specimens after ablation tests.

## 3. Results and Discussion

The values of the densities of the manufactured CFRE-Cs are presented in Table 1. The results specified that the addition of NDs in the epoxy composites caused a slight reduction in the density of the composite specimens. Even though the decrease was not very substantial, it can be deduced that the incorporation of nanofiller replaced the area that was initially occupied only by the neat epoxy matrix and caused some porosity [20].

**Figure 5:**
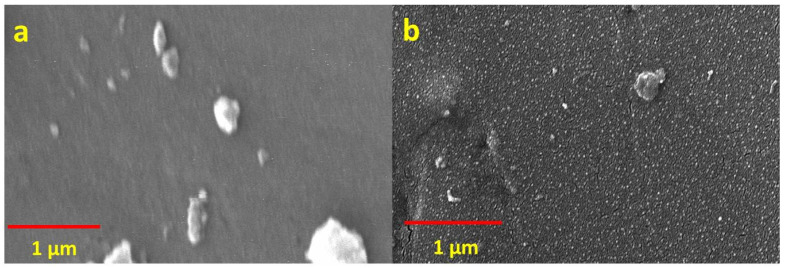
SEM images of CF–epoxy composites with and without NDs. (**a**) Neat epoxy matrix; (**b**) epoxy matrix containing NDs.

Figure 5 shows SEM images of the as-manufactured CF–epoxy matrix composites with and without NDs. The epoxy matrix without NDs is evident in Figure 5a. Furthermore, Figure 5b also exposes the presence of uniformly distributed NDs in the epoxy matrix, as achieved by their functionalization [21,22].

**Figure 6:**
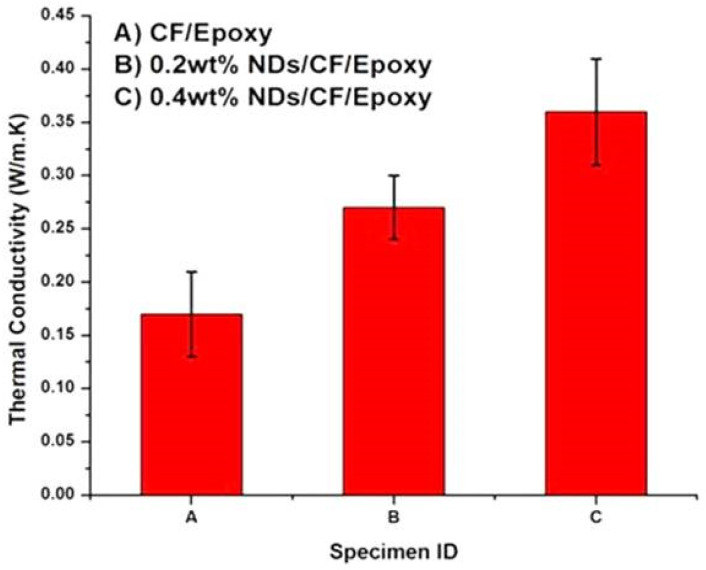
Thermal conductivity values of (**A**) CFRE-C, (**B**) CFRE-C containing 0.2 wt% NDs, and (**C**) CFRE-C containing 0.4 wt% NDs.

The thermal conductivity values of the composite specimens are presented in Figure 6. It can be perceived from the graph that the thermal conductivity of CFRE-C without nanofillers was around 0.17 W/m.K, which was improved to 0.27 W/m.K by incorporating 0.2 wt% NDs in CFRE-Cs and by incorporation of more quantity of NDs, i.e., 0.4 wt% NDs, an enhancement of 0.36 W/m.K was witnessed. The rise in the thermal conductivity performance of the CFRE-Cs was a result of the addition of NDs, which inherently possess high thermal conductivity characteristics [23,24].

**Figure 7:**
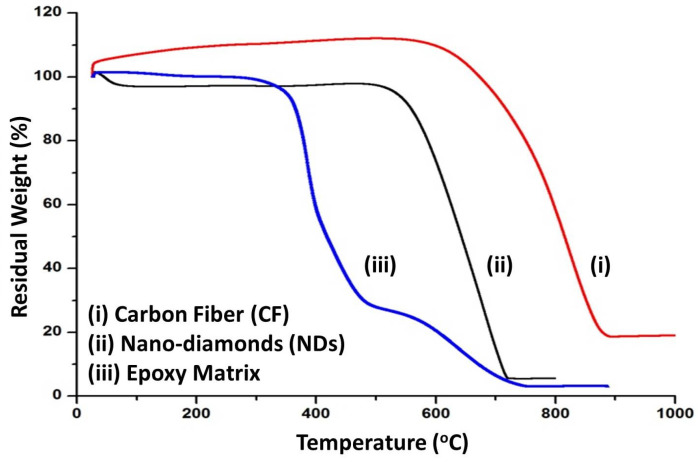
TGA curves depicting the thermal decomposition of epoxy, NDs, and CFs at different temperatures.

Results from the thermogravimetric analysis of the components utilized to manufacture the CFRE-Cs, i.e., neat epoxy, NDs, and CFs, are shown in Figure 7. It can be observed that Araldite^®^ 5052 epoxy resin revealed a two-step weight-loss curve. After the initial weight loss due to hydration, the first step of weight loss took place at 380 °C followed by weight loss at 580 °C and leaving behind a residual char yield of 3.6%, which is the consequence of the creation of carbonaceous compounds due to chemical networking of the epoxy resin, whereas, the second step implicates the thermo-oxidative response conforming to the whole degradation of carbonaceous materials at 1200 °C. Decomposition of NDs displayed a single-step weight loss curve at the oxidation temperature of ~590 °C. The degradation process continued till a temperature of 720 °C was reached, at which point the carbonaceous materials comprising diamond, graphite, and amorphous carbon were oxidized. Contrary to the preceding degradation profiles, CF exhibited a single-step degradation curve with an onset temperature of degradation at 700 °C.

**Figure 8:**
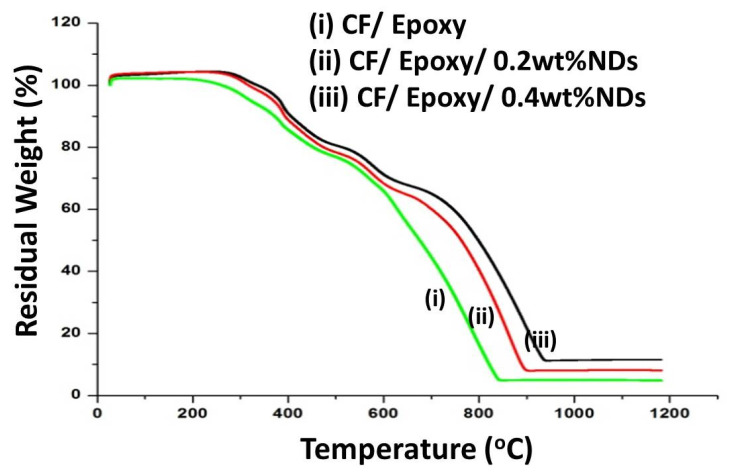
TGA curves depicting thermal response of (i) CF/epoxy composites (ii) CF/epoxy/0.2 wt% NDs, and (iii) CF/epoxy/0.4 wt% NDs.

Results from the thermogravimetric analysis of composite samples with and without NDs are shown in Figure 8. The composite sample without NDs showed a two-step weight loss curve which corresponds to the epoxy and the CF. The thermal profile of CF composites containing NDs showed a three-step degradation profile with an increase in the thermal stability of the composites. The residual weight percentage of the composites is presented in Table 2. It can be observed from the degradation profiles after 1000 °C that the residual content of the composites increased with the increase in ND content. Hence, it’s evident that increasing the ND content in CFRE-Cs increases the thermal stability.

**Figure 9:**
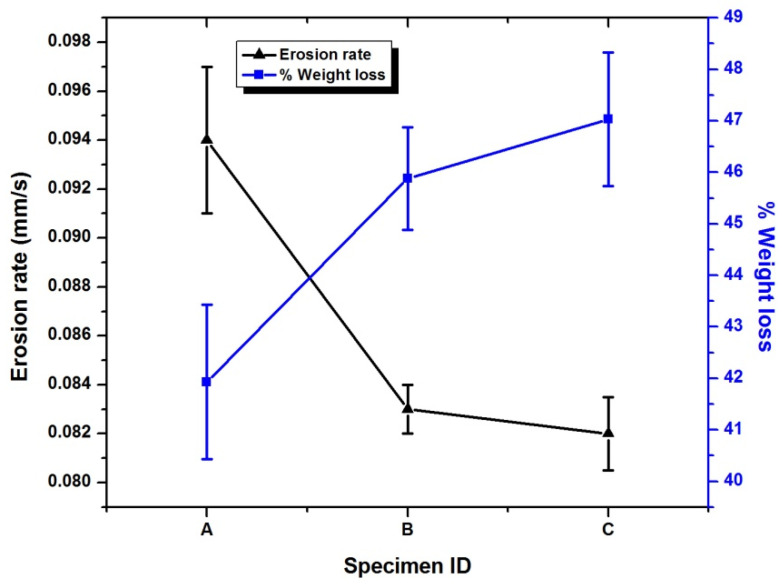
Percentage weight loss and erosion rate of CFRE-Cs with and without NDs. Specimen A represents CF/epoxy composite, specimen B represents CF/epoxy/0.2 wt% NDs composite, and specimen C represents CF/epoxy/0.4 wt% NDs composite.

Weight loss and the erosion rate calculations of CFRE-Cs are displayed in Figure 9. The calculated value of erosion of composite without NDs was 0.94 ± 0.03 mm/s which is almost close to the CF phenolic resin composites value [5] with a char yield of more the 50%. The addition of 0.2 wt% NDs in CFRE-C decreased the 10.5% of erosion rate, which further reduced by 12.6% with the 0.4 wt% NDs sample. It was also verified to be useful in ablation as the weight loss after ablation was reduced with an increase in the ND content.

**Figure 10:**
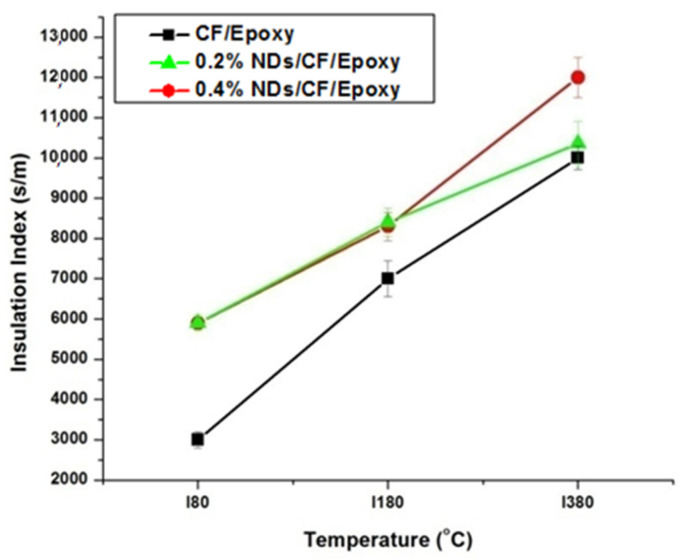
Insulation index performance of CFRE-C specimens at temperatures 80 °C, 180 °C, and 380 °C.

**Figure 11:**
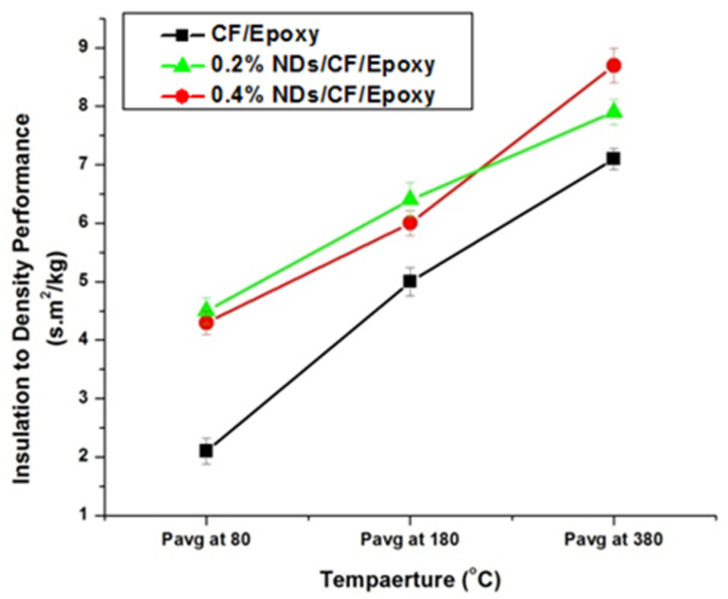
Insulation-to-density values of CFRE-C specimens at temperatures 80 °C, 180 °C, and 380 °C.

The insulation index ($I_{T}$), and insulation-to-density performance index ${\left(P_{avg}\right)}_{T}$ were determined by utilizing the succeeding terms, and the calculated results are shown in Figure 10 and Figure 11, respectively.

(1) $$I_{T}=\frac{t_{T}}{d}$$

where $t_{T}$ represents the time for back-face temperature rises to 80 °C, 180 °C, and 380 °C, and d is the thickness of the specimen.

(2) $${\left(P_{avg}\right)}_{T}=\frac{{\left(I_{T}\right)}_{avg}}{D_{avg}}$$

where ${\left(I_{T}\right)}_{avg}$ represents the average insulation index at temperature (T) and $D_{avg}$ represents theaverage density of the specimen.

The insulation indexes measured for the composite samples at different temperatures are presented in Figure 10. The insulation index values of CFRE-Cs without NDs is comparatively low compared to the CFRE-Cs containing 0.2 wt% NDs at low temperatures, contributing to an increase in thermal conductivity of CFRE-Cs. Enhancement in the thermal conductivity performance of the material led to an enhanced influence of heat and resulted in higher insulation index values. The insulation index values of specimens comprising 0.2 wt% and 0.4 wt% NDs were the same at lower temperatures; nevertheless, at higher temperatures, i.e., 360 °C, the value of the composite specimen containing 0.4 wt% NDs become considerably higher than the composite specimen containing 0.2 wt% NDs, which may be attributed to comparatively less thermal conductivity, less affected area, and less thermal stability of the specimen containing 0.2 wt% NDs.

Insulation-to-density performance measurements of the composite samples are displayed in Figure 11. The insulation-to-density performance of 0.4 wt% ND-loaded composite specimens was higher as a result of their higher insulation indexes. At higher temperature values, the composite specimens comprising 0.4 wt% NDs revealed an optimal performance. Nevertheless, the lowest values were measured for the composites without NDs.

**Figure 12:**
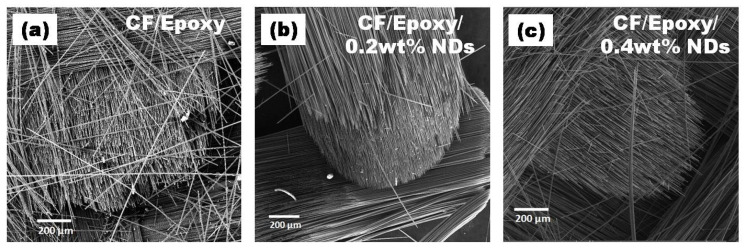
SEM images of CFRE-C (**a**) fiber fracture and (**b**,**c**) curved fiber toes due to uniform burning.

**Figure 13:**
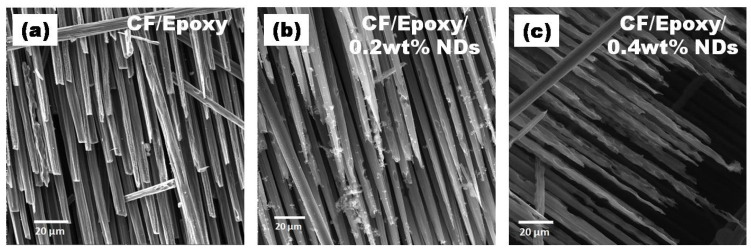
SEM images of CFRE-Cs: (**a**) blunt edges of fiber in CFRE-Cs, (**b**) sharp edges of fibers in 0.2 wt% NDs/CFRE-Cs, and (**c**) surface pitting on fibers in 0.4 wt% NDs/CFRE-Cs.

**Figure 14:**
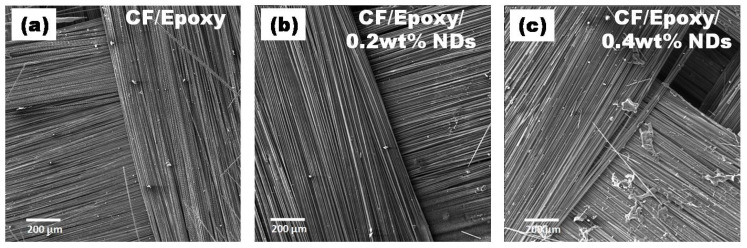
SEM images of CFRE-C 7 mm away from flame hitpoint where (**a**) CF/Epoxy, (**b**) CF/Epoxy/0.2 wt% NDs and (**c**) Cf/Epoxy/0.4 wt% NDs.

**Figure 15:**
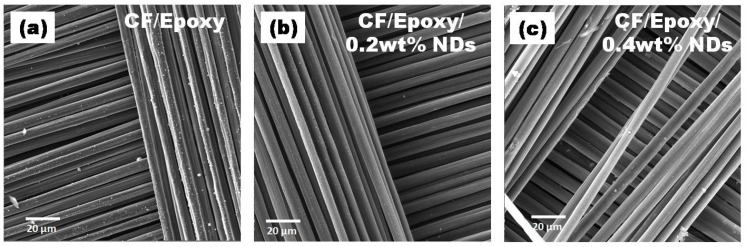
Magnified SEM images of CFRE-Cs 7 mm away from flame hit point where (**a**) CF/Epoxy, (**b**) CF/Epoxy/0.2 wt% NDs and (**c**) Cf/Epoxy/0.4 wt% NDs.

SEM images of surfaces after ablation tests at various amplifications are presented in Figure 12, Figure 13, Figure 14 and Figure 15. Disruption of fiber was found to be widespread in CFRE-Cs (Figure 12a) in comparison to composites containing NDs, as revealed in Figure 12b,c, where ablation can be seen uniformly deprived of heavy fragments of fibers. The ablated areas of the CFRE-Cs were studied at high magnifications to comprehend the ablation behavior of NDs incorporated in CFRE-Cs. Blunt fiber tips of CFRE-C without NDs (Figure 13a) revealed that the ablation was less influenced because of the radial recession of fibers across the length. It was primarily because of oxidation and mechanical erosion of fibers where the flame directly hit and rose to push apart the fibers within tows. Low thermal conductivity [25] may have improved the thermal influence at the localized area. Consequently, an even radial recession of needle-like fibers was not detected, representing a characteristic feature of CFs in C-C composites [26] and witnessed in composites comprising NDs (Figure 13b,c). Composites having 0.4 wt% NDs exhibited extreme thinning of fibers across more considerable lengths than the composites containing 0.2 wt% NDs. In addition, the high thermal conductivity of NDs enhanced the thermal properties of the epoxy matrix by incorporating NDs in the neat epoxy matrix to form a modified epoxy matrix. This conductivity effect produced the heat flow in a more significant volume and increased the heat-affected area, causing absorption of additional heat flux. SEM images of CFRE-Cs at a point ~7 mm away from the ablated area displayed un-ablated CFs with residual epoxy and oxidation products (Figure 14). By further magnifying these regions (Figure 15), the presence of oxidation products was detected on the surface of CFs in abundance in composites containing NDs compared to reference composites, i.e., CFRE-Cs without NDs. Similarly, more carbonaceous products were found on composite specimens containing 0.4 wt% NDs that were not present on composites containing 0.2 wt% NDs (Figure 14b,c, validating the results of TGA (Table 2), producing more char compared to reference CFRE-Cs.

The phenomena of ablation in CFRE-Cs comprising NDs is generally a surface ablation phenomenon [27] instead of a volume phenomenon. The epoxy matrix was thermoset sublimed instead of transforming into a liquid phase like thermoplastics at a particular temperature and leaving behind carbonaceous char and CFs that possess a high sublimation temperature of 2000 °C [28,29]. By introducing NDs (third phase) in CFRE-Cs, further enhancement in the recession resistance of the CFRE-C specimens during ablation testing was witnessed. Therefore, it is evident that the incorporation of NDs led to escalating the thermal stability and thermal conductivity performance of CFRE-Cs. Consequently, erosion resistance of CFRE-Cs was also enhanced because of increased thermal stability and thermal conductivity of CFRE-Cs (Figure 6). The subsequent increase in thermal conductivity brought more volume to be affected due to heat of ablation, which resulted in oxidation of further CFs and consumption of more heat. The enhancement in the thermal performance of CFRE-Cs can also be co-related with increased weight loss of CFRE-Cs’ material (Figure 9). With an increase in NDs up to 0.4 wt%, the thermal conductivity upsurged, leading to optimal erosion resistance.

**Figure 16:**
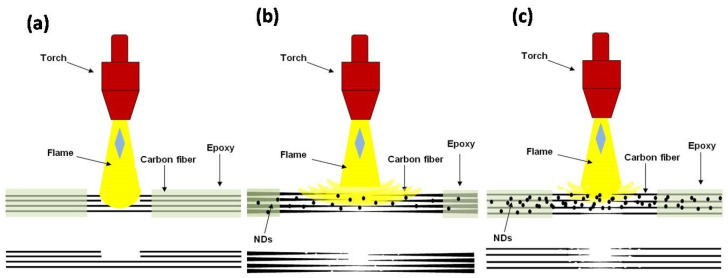
Mechanism of ablation: (**a**) CF–epoxy composites without NDs, (**b**) CF–epoxy composites containing 0.2 wt% NDs, and (**c**) CF–epoxy composites comprising 0.4 wt% NDs.

The schematic diagrams of mechanisms observed while performing ablation tests of three different types of CFRE-Cs are presented in Figure 16. Figure 16 ademonstrates the ablation phenomena as observed in the manufactured CFRE-Cs without NDs. It is evident in the diagram that because of having a thermally insulating property, the epoxy matrix may resist the transmission of heat efficiently in the bulk of the material, and, therefore, restrict heat flux on a small area where the flame was intact. As a result, a small, focused area erosion of CFs was witnessed along with blunt edges of the CFs because of the point focused and elevated temperature of the flame and optimum shear force generated by the high velocity of the flame. Figure 16b illustrates the ablation performance of CFRE-Cs comprising 0.2 wt% NDs. The incorporation of NDs in the epoxy matrix of CFRE-Cs assisted in the enhancement of thermal conductivity of the epoxy matrix, which ultimately caused the heat flux to expand to more areas in composite specimens (Figure 13b). The needle-like morphology of CFs after ablation and increased size of penetration of the flame supports the theory of increased affected area via increased thermal conductivity. By increasing the quantity of NDs from 0.2 wt% to 0.4 wt%, the needle-like morphology became more pronounced and through-thickness permeation became more challenging due toincreased affected area and increased fiber-thinning lengths (Figure 16c).

## 4. Conclusions

The study delivered details about the influence of different amounts of NDs on the thermal and ablative performance of manufactured CFRE-Cs. Thermal conductivity values of CFRE-Cs were amplified to 37% and 52% by introducing 0.2 wt% and 0.4 wt% NDs, respectively, in the epoxy matrix, causing an increase in the enlarged heat affected area as well and an enhancement in the erosion resistance by 10.5% and 12.6% with 0.2 wt% and 0.4 wt% NDs, respectively. The erosion rate was influenced by weight loss after ablation testing, which further revealed that the addition of NDs led to improved weight loss because of the increased heat affected area due to the high thermal conductivity performance of CFRE-Cs. Furthermore, the needle-like shape of CFs was witnessed while performing SEM, which validated the high-heat-affected area being extended through more extended lengths of fiber, which may be accredited to the enhanced thermal conductivity performance of CFRE-Cs because of the incorporation of NDs in the epoxy matrix.

## Figures and Tables

**Table 1:** Measured and relative density values of the CFRE-C specimens with and without NDs.

Composites	Density (g/cm^3^)	Relative Density (%)
CF/epoxy	1.40 ± 0.02	96 ± 0.2
0.2 wt% NDs/CF/epoxy	1.37 ± 0.03	95 ± 0.2
0.4 wt% NDs/CF/epoxy	1.37 ± 0.03	95 ± 0.2

**Table 2:** Residual weight of composites at different temperatures.

Temperature (°C)	Residual Weight (%)		
	CF/Epoxy	0.2 wt% NDs/CF/Epoxy	0.4 wt% NDs/CF/Epoxy
500	76.85	78.34	80.56
600	65.97	68.37	71.22
700	44.40	60.13	65.03
1200	4.73	8.02	11.47

## Data Availability

The data presented in this study are available on request from the corresponding author.
